# A combined clinical and specific genes’ model to predict live birth for in vitro fertilization and embryo transfer patients

**DOI:** 10.1186/s12884-023-05988-6

**Published:** 2023-09-30

**Authors:** Shihui Meng, Cheng Shi, Yingying Jia, Min Fu, Tianzhen Zhang, Na Wu, Hongjing Han, Huan Shen

**Affiliations:** 1 Department of Obstetrics and Gynecology, Beijing Tiantan Hospital, Capital Medical University, Beijing, China; 2 Reproductive Medical Center, Department of Obstetrics and Gynecology, Peking University People’s Hospital, Peking University, Beijing, 100044 China; 3 Department of Central Laboratory and Institute of Clinical Molecular Biology, Peking University People’s Hospital, Beijing, China

**Keywords:** Endometrial receptivity, Gene expression, Ovulation dysfunction, LASSO regression, Live birth

## Abstract

**Background:**

We aimed to develop an accurate model to predict live birth for patients receiving in vitro fertilization and embryo transfer (IVF-ET) treatment.

**Methods:**

This is a prospective nested case–control study. Women aged between 18 and 38 years, whose body mass index (BMI) were between the range of 18.5–24 kg/m^2^, who had an endometrium of ≥ 8 mm at the thickest were enrolled from 2018/9 to 2020/8. All patients received IVF-ET treatment and were followed up until Jan. 2022 when they had reproductive outcomes. Endometrial samples during the window of implantation (LH + 6 to 9 days) were subjected to analyze specific endometrial receptivity genes’ expression using real-time PCR (RT-PCR). Patients were divided into live birth group and non-live birth group based on IVF-ET outcomes. Clinical signatures relevant to live birth were collected, analyzed, and used to establish a predictive model for live birth by univariate analysis (clinical model). Specific endometrial receptivity genes’ expression was analyzed, selected, and used to construct a predictive model for live birth by The Least Absolute Shrinkage and Selection Operator (LASSO) analysis (gene model). Finally, significant clinical factors and genes were used to construct a combined model for predicting live birth using multivariate logistical regression (combined model). Different models’ Area Under Curve (AUC) were compared to identify the most predictive model.

**Results:**

Thirty-nine patients were enrolled in the study, twenty-four patients had live births, fifteen did not. In univariate analysis, the odds of live birth for women with ovulation dysfunction was 4 times higher than that for women with other IVF-ET indications (OR = 4.0, 95% CI: 1.125 − 8.910, *P* = 0.018). Age, body mass index, duration of infertility, primary infertility, repeated implantation failure, antral follicle counting, ovarian sensitivity index, anti-Mullerian hormone, controlled ovarian hyperstimulation protocol and duration, total dose of FSH/hMG, number of oocytes retrieved, regiment of endometrial preparation, endometrium thickness before embryo transfer, type of embryo transferred were not associated with live birth (*P* > 0.05). Only ovulation dysfunction was used to construct the clinical model and its AUC was 0.688. In lasso analysis, GAST, GPX3, THBS2 were found to promote the risk of live birth. AUCs for GAST, GPX3, THBS2 reached to 0.736, 0.672, and 0.678, respectively. The gene model was established based on these three genes and its AUC was 0.772. Ovulation dysfunction, GAST, GPX3, and THBS2 were finally used to construct the combined model, reaching the highest AUC (AUC = 0.842).

**Conclusions:**

Compared to the single model, the combined model of clinical (Ovulation dysfunction) and specific genes (GAST, GPX3, THBS2) was more accurate to predict live birth for IVF-ET patients.

**Supplementary Information:**

The online version contains supplementary material available at 10.1186/s12884-023-05988-6.

## Background

In vitro fertilization (IVF) has become a standard method for treating infertility. Despite its many advances, the current live birth rates were 25–30% per started cycle [1]. Over the years, prediction models with clinical factors have been developed to help tailoring treatment protocols and to provide guidance in clinical choice. Clinical factors including obstetrical treatment history, physical examination, infertility work-up were used to construct models. Models have been shown to be effective in preserving high pregnancy rates and live birth rates [2]. However, with the development of endometrial sequencing, endometrial RNA sequencing data with artificial intelligence (AI) led to a clinical revolution in personalizing endometrial receptivity diagnosis. The endometrial receptivity array (ERA) consists of 238 genes expressed at the different stages of the endometrial cycle to classify patients as non-receptive endometrium, either pre- or post-receptive, and receptive endometrium regardless of the sample's histologic appearance [3]. The results of ERA did not differ significantly in samples from the same patient at the same menstrual period and were reproducible in the same patients 29–40 months after the first test [4]. In the non-receptive phase, the clinical outcome was 23% pregnancy rate and 13% implantation rate after transfer, with 0% ongoing pregnancy rate. In contrast, embryo transfer during receptive phase can achieve 60% pregnancy rate, 45% implantation rate and 74% ongoing pregnancy rate [5]. After ERA diagnosis, patients can reach a 60% clinical pregnancy rate in a receptive endometrium [4]. RIF patients can achieve 42.4% ongoing pregnancy rate after personalized embryo transfer, showing the diagnostic and therapeutic value of ERA [6]. However, most transcriptomic profiles were based on histology dating and focused on gene differences between receptive and non-receptive phases during the same menstrual cycle. The different RNAs expression during window of implantation between patients who achieve live birth after IVF-ET and who do not need further study. Therefore, live birth between patients undergoing routine embryo transfer and those who following personalized embryo transfer remains unknown. Furthermore, there has been much debate and paucity of available data regarding the impacts of this test on the clinical outcomes of embryo transfer in patients undergoing IVF, and this is still a poorly investigated and controversial area [7].

We aimed to construct a new predictive model for IVF-ET patients’ live birth based on clinical and specific genes’ expression of endometrium during the window of implantation (WOI). RT-PCR were used to detect the genes’ expression, and different statistical methods were used to construct various models. Finally, the accuracy of each model was compared to find the most suitable model.

## Methods

### Patients selection and study design

This study enrolled infertile patients who met all the inclusion criteria: women aged between 18 and 38 years, body mass index (BMI) were between the range of 18.5–24 kg/m^2^, had an endometrium of ≥ 8 mm at the thickest, planned to receive in vitro fertilization and frozen embryo transfer. Women with one or more of the following conditions were excluded: Patients did not have morphology good embryo before embryo transfer, had hysteromyoma, fibroids, endometriosis, intrauterine adhesion. Definition of morphology good embryo was 8–10 cell stage, ≤ 15% fragmentation, even cellular cleavage for cleavage-stage embryos; for the blastocyst, the inner cell mass was prominent, easily discernible, with many cells that are compacted and tightly adhered together, the trophectoderm should have many cells forming a cohesive epithelium. Ethics approval for this trial was obtained from the ethics committee of the Peking University People’s hospital (2018PHB141-01). Patients were enrolled between Sep 8, 2018, and Aug 19, 2020. The follow up was finished in January 2022. A signed informed consent form was obtained from all patients.

Data were prospectively collected before and during IVF-ET treatment. Obstetrical and treatment history were self-reported. Patients underwent either a natural cycle or hormone replacement therapy cycle to receive endometrial biopsy.

### Endometrial biopsy specimens

In a natural cycle, patients performed ovulation tests to determine the luteinizing hormone (LH) surge using semiquantitative, urine-based commercial kit (Eupregna). Patients received endometrium biopsy during 7 to 9 days after LH surge with blood progesterone < 1.5 ng/ml. In the artificial cycle, endometrial biopsy was performed 5–9 full days of progesterone impregnation with endometrium measuring ≥ 8 mm.

Endometrial biopsy samples were obtained with dilation disposable uterine-cavity tissue-suction tubes (Jingyou SAP-I). Each sample was divided into two portions. One was fixed in 10% formalin and processed for histological evaluation (hematoxylin–eosin, H–E) to check whether it was in mid-luteal phase. The second portion was stored at liquid nitrogen for RNA extraction.

### IVF-ET treatment

All patients received standardized ovarian stimulation regimens, oocyte retrieval, and fertilization, followed by a planned frozen transfer of up to two embryos of day-3 or day-5. Patients received one of the following regimens at the discretion of local investigators: gonadotropin-releasing–hormone (GnRH) antagonist, GnRH-agonist long protocol, mild stimulation protocol. When at least one follicle reached 18 mm, 5,000 to 10,000 IU of hCG (Covidrel, Merck Serono) was administered and oocyte retrieval occurred 36 h later.

The cleavage embryo morphology was recorded on day 3 based on the scoring system reported by Puissant et al [8]. Briefly, the number and evenness of the blastomeres were analyzed, as well as the fragment percentages. Cleaved embryos with 7–10 equal or slightly unequal blastomeres and ≤ 15% fragments were considered as grade I. When the percentage of fragments was 16%–29% or the number of blastomeres did not meet the grade I standard, the embryos were considered as grade II. When the percentage of fragments was between 30 and 49%, the embryos were considered as grade III. Finally, when there were 50% fragments or the embryo development was retarded, the embryos were considered as grade IV. Usually, two embryos at grade I or II were selected for freezing on day 3, and the others were group cultured in blastocyst medium for another 2 or 4 days. Day 5, 6 or 7 blastocysts were morphological recorded and classified into three grades according to the expansion state of blastocele, the quality of inner cell mass and trophectoderm cells. Only good or median blastocysts (at least trophectoderm or inner cell mass score ‘B’ or ‘A’) were frozen. In the thawing cycle, cryopreserved blastocysts were thawed for transfer in priority. After all the cryopreserved blastocysts were used and none was left, or there were not blastocysts cryopreserved, cryopreserved cleavage embryos were thawed for transfer. For blastocyst thawing cycle, one blastocyst was thawed for transfer if the patients were young (< 35 y) and transferred for the first time. However, if patients aged ≥ 35 y and had failed embryo transfer history, two blastocysts could be transferred. For cleavage embryo thawing cycle, two embryos were usually transferred considering the low successful rate for cleavage embryos. The treatment protocol used for embryo transfer is the same as used during the biopsy cycles for each patient. In natural cycle, luteal-phase support was started from the day of ovulation with oral dydrogesterone at a dose of 10 mg three times a day and was continued until the day of serum hCG testing. In hormone replacement therapy, estradiol tablet was started 6 mg per day until endometrium reached at least 8 mm, when 40 mg progesterone injection was used on P + 0, and 60 mg progesterone injection was used from P + 2 to P + 3/P + 5 based on the day of embryo. Pregnancy test will be performed two weeks after embryo transfer. In women with a positive hCG test, luteal phase support was continued until 10 weeks of gestation. Live birth, which was defined as the delivery of any viable neonate who was 28 weeks of gestation or older, was followed up. Patients were divided into two groups based on whether they had live birth: live birth group and without live birth group. All data were prospectively collected before and during IVF/ICSI treatment, and the resulting effects were recorded. All seventeen variables that were collected are presented in Supplemental Table 3.

### RNA extraction, purifying and quantitative real-time PCR

Total RNA was isolated from the endometrial specimens using Trizol reagent (Invitrogen, Waltham, MA). The purity and concentration of RNA was determined by OD260/280 (NanoDrop, ND-1000). RNA integrity was examined by 1% formaldehyde denaturing gel electrophoresis. RNA with an OD260/280 between 1.8 and 2.0 and no degradation by electrophoresis was used for RT-PCR experiments.

We did quantitative real-time PCR analysis on genes from our previous study and classical genes reported relevant to endometrial receptivity: CXCR4, DHRS3, DPP4, GAST, GPX3, HABP2, HEY2, IGFBP1, LEPREL, MAP2K6, PROM1, SERPING, SFRP4, THBS2, TIMP3, TNFAIP2, MUC1, HOXA10, GPR110, LIF, L-selectin, FKBP52, HAND2 (Primer sequences see Supplemental Table 1). About 5ug of total RNA from each sample was used for RT reaction to generate cDNA using Prime Script reagent Kit with gDNA Eraser (TaKaRa, Tokyo, Japan). Quantitative real-time PCR was performed in triplicate using SYBR Premix Ex Taq ™ II (Tli RNaseH Plus), ABI7500 (Applied Biosystems, Foster City, CA) and ROX plus (TaKaRa, Tokyo, Japan) according to the instructions from the manufacturer. We monitored a single PCR product and the absence of primer dimers by melting curves. The data were normalized to the expression levels of the housekeeping gene β-ACTIN, and relative expression was calculated using 2^–DDCt^.

### Statistical analysis

Continuous variables are given as median with interquartile range (IQR), and categorical variables are given as frequency or percentage. First, univariate analysis was performed by entering live birth as a binary variable in the models to construct the clinical model. Age, body mass index, ovarian sensitivity index, anti-Mullerian hormone, antral follicle counting, duration of COH, total dose of FSH/hMG, number of oocytes retrieved, endometrium thickness before embryo transfer were analyzed as continuous variables. Stratified analyses were conducted according to indications for IVF, controlled ovarian hyperstimulation protocol, regiment of endometrial preparation, and type of embryo transferred. The t-test and the chi-square test or Mann–Whitney U test was used in baseline characteristics. Variables with P values < 0.05 were selected for further analysis.

Gene model was constructed by LASSO regression to select the most relevant and interpretable set of gene predictors from a large and potentially multicollinear set of variables in the regression. According to McEligot AJ et al., we utilized the “glmnet” package (version 2.0–16) to fit the logistic LASSO regression [9]. The selected genes resulting from LASSO regression analysis were used to construct a risk score signature. We utilized three-fold cross-validation to select the penalty term, λ. The built-in function in R produces two automatic λ’s—one that minimizes the binomial deviance and one representing the largest λ that is still within 1 standard error of the minimum binomial deviance. We opted for the latter λ as it results in a stricter penalty allowing us to reduce the number of covariates even further than the former λ. The risk score based on the signature was calculated by the following formula [10]:

$$\mathrm{Risk score}=\sum {\mathrm{n}}_{\mathrm{i}}=\sum (\mathrm{Coefi}*{\mathrm{x}}_{\mathrm{i}})$$

Coefi was the coefficient and x_i_ was the z-score transformed relative expression value of each selected gene. ROC curve for each gene selected from LASSO regression and the constructed gene model were demonstrated. The predictive or prognostic accuracy was indicated by the area under the ROC curve (AUC).

The combined model was constructed by using significant clinical factors and genes in both the clinical model and gene model. Multivariable logistic regression analysis was applied and the risk score based on the signature was calculated by the following formula:

$$\mathrm{Risk score}=\sum {\mathrm{n}}_{\mathrm{i}}=\sum (\mathrm{Coefi}*{\mathrm{x}}_{\mathrm{i}})$$

## Results

### Characteristics of patients

**Fig. 1:**
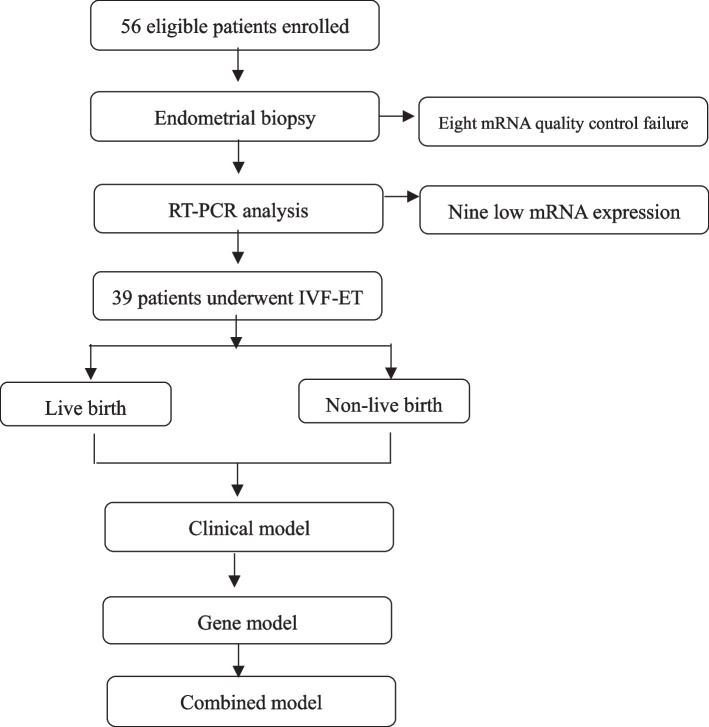
The flow chart of the study design and analysis

A total of 56 patients were enrolled, endometrium samples were collected, and finally 39 samples were analyzed successfully by RT-PCR (Fig. 1). All 39 patients’ demographic and treatment cycle characteristics are shown in Table 1. Twenty-four patients had live birth, 15 patients did not, live birth rate of all patients was 61.5%. Age, ovulatory factor, controlled ovarian hyperstimulation protocol, total dose of FSH/hMG were different between patients with live birth and patients without live birth.

**Table 1:** Characteristics of patients with endometrium samples

Variable	**Patients with live birth** ***N* = 24**	**Patients without live birth** ***N* = 15**	***P*-value**
**Age, y**	32.5(28.3,37.0)	32(29,35)	*P* = 0.028, t = 0.4
**Body mass index, kg/m**^**2**^	23.1(21.2,23.4)	22.9(20.7,23.9)	*P* = 0.4, t = -0.09
**Fertility history**			
Duration of infertility, year	3(2,4)	2(1,3)	*P* = 0.2, t = 0.25
Primary infertility, no. (%)	16(66.7)	8(53.3)	*P* = 0.41, χ^2^ = 0.69
**Indications for IVF, no. (%)**			
Endometriosis	1(4.1)	1(6.7)	*P* = 0.73, χ^2^ = 0.12
Tubal factor	12(50)	7(46.7)	*P* = 0.84, χ^2^ = 0.04
Male factor	18(75)	11(73.3)	*P* = 0.91, χ^2^ = 0.01
Ovulatory factor	7(33.3)	2(13.3)	*P* = 0.02, χ^2^ = 8.41
**Number of failed embryo transfer cycle, median (range)**	0.63(0–3)	1.28(0–8)	*P* = 0.03, t = -1.04
**Repeated implantation failure**, **no. (%)**	2(40)	3(60)	*P* = 0.29, χ^2^ = 1.12
**Hormone tests and Ultrasound**			
AMH, ng/ml	3.85(1.83,6.02)	4.31(2.47–7.5)	*P* = 0.33, t = -0.72
Antral Follicle Counting	14(7.5,19.8)	11(10,17)	*P* = 0.67, t = -0.12
Ovarian sensitivity index (OSI)	0.79(0.51, 0.90)	0.80(0.48, 1.01)	*P* = 0.82, t = -0.54
**COH protocol**			
GnRH agonists, no. (%)	15(65.2)	8(34.8)	*P* = 4 × 10^–3^, χ^2^ = 10.98
GnRH antagonists, no. (%)	7(29.2)	7(46.7)	
CC Mild stimulation, no. (%)	2(100)	0(0)	
**Duration of COH, (day)**	10(9.25,12)	11(9, 12)	*P* = 0.46, t = -0.20
**Total dose of FSH/hMG (UI)**	2306.25(1968.75, 2925)	2700(1762.5,3600)	*P* = 0.02, t = -0.94
**No. of oocytes retrieved**	13.5(8, 16.75)	15(11, 17)	*P* = 0.13, t = -0.56
**Endometrial thickness before ET**	9.5(9,11.3)	8(7.8,10.5)	*P* = 0.22, t = 1.53
**Regiment of endometrial preparation**			
Natural cycle no. (%)	15(62.5)	9(60)	*P* = 0.88, χ^2^ = 0.02
Artificial cycle no. (%)	9(37.5)	6(40)	
**Luteal-phase support**			
Oral dydrogesterone protocol	15(62.5)	9(60)	*P* = 0.88, χ^2^ = 0.02
Progesterone injection protocol	9(37.5)	6(40)	
**Number of embryos transferred**			
One embryo	4(16.7)	5(33.3)	*P* = 0.27, χ^2^ = 1.45
Two embryos	20(83.3)	10(66.7)	
**Type of embryo transferred**			
Cleavage transfer, no. (%)	13(54.2)	4(26.7)	*P* = 0.09, χ^2^ = 2.84
Blastocyst transfer, no. (%)	11(45.8)	11(73.3)	

*AMH* Anti-Mullerian Hormone, *COH* Controlled ovarian stimulation, *ET* Embryo transfer

### Impact of each clinical variable on clinical pregnancy by univariate analysis

**Fig. 2:**
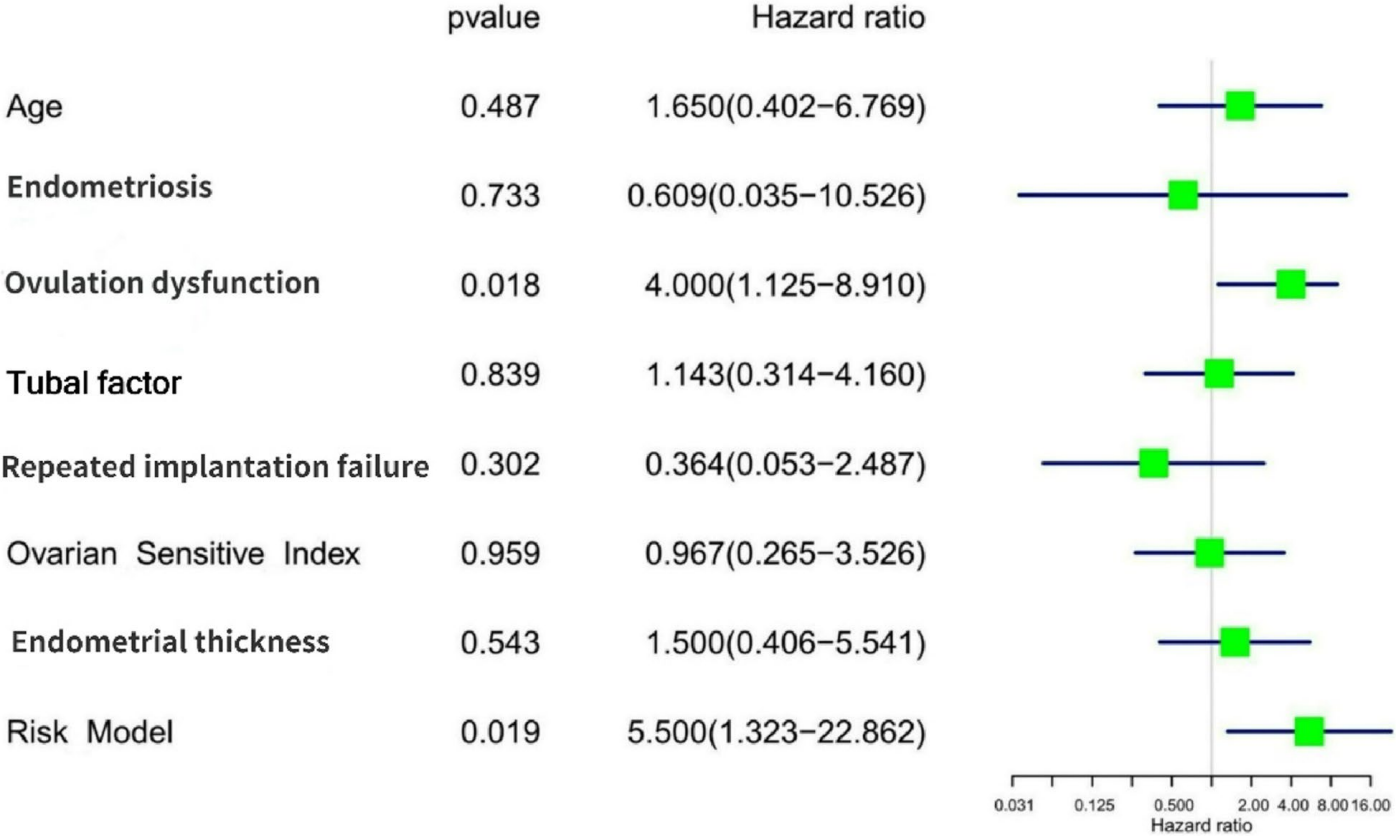
Results of univariate analysis of clinical features and live birth. Ovulation dysfunction was associated with live birth. Ovarian sensitivity index = log (number of eggs recovered × 1,000/total dose of FSH)

As determined by univariate analysis, ovulation dysfunction (Fig. 2) was associated with live birth. The odds of success with IVF treatment for women with ovulation dysfunction was 4 times higher than for women with other indications. Ovarian sensitivity index (OSI) is a composite variable to measure ovarian response. It is derived with the use of the formula: OSI = log (number of eggs recovered × 1,000/total dose of FSH). It is was not correlated with live birth in the univariate analysis. The result of unsignificant variables were shown in Supplemental Table 3.

### Relationship between live birth and gene features

**Fig. 3:**
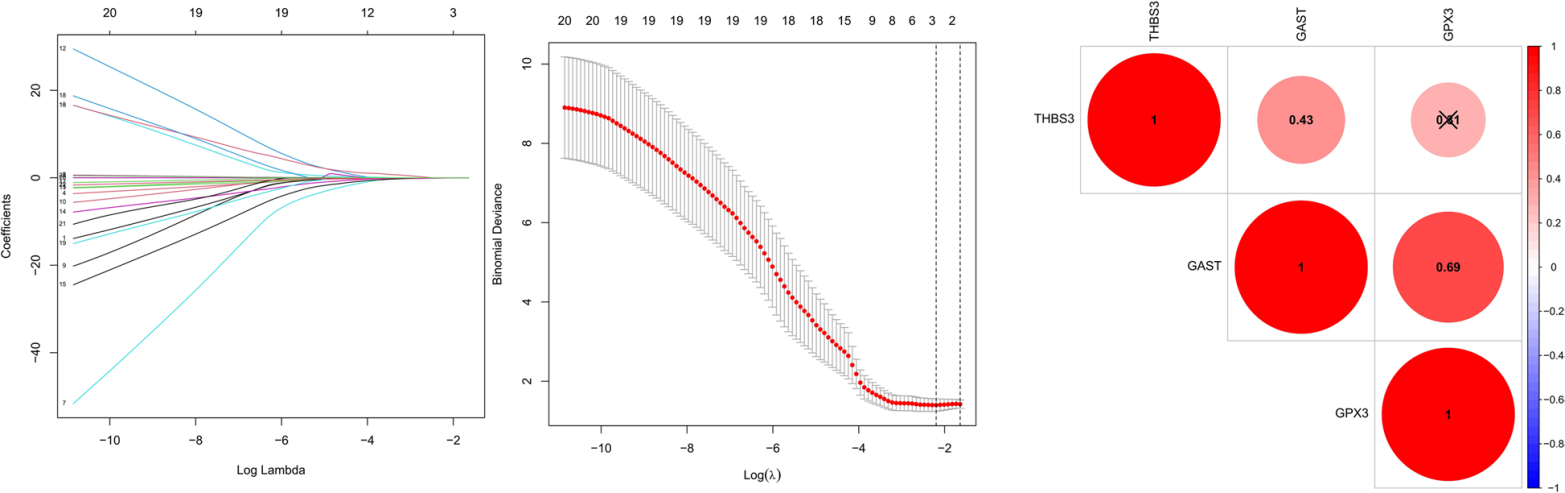
The process of selecting targeted genes by LASSO regression model. The LASSO analysis includes 21 genes. When log lambda ranges from -1.0 to -3.0, three genes: GAST, GPX3, THBS2, were selected

**Fig. 4:**
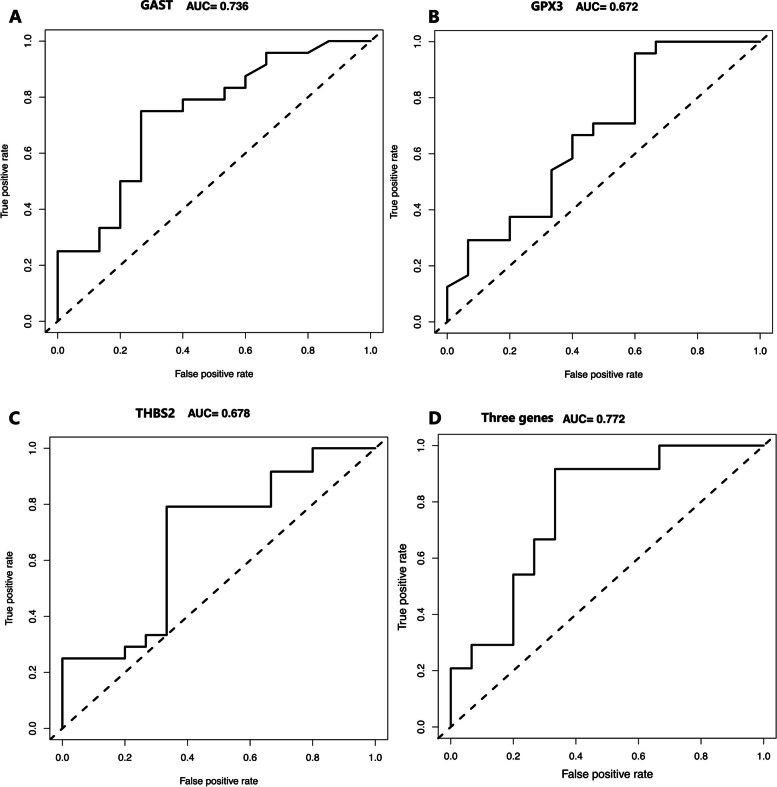
ROC curves for individual predictive genes and combined genes associated with live birth. AUC of single gene (AUC _GAST_ = 0.736, AUC_GPX3_ = 0.672, AUC_THBS2_ = 0.678) was lower than combined genes model (AUC = 0.772) in predicting live birth

The mRNA expression during the window of implantation was shown in Supplemental Table 2. To investigate the relationship between mRNA expression and live birth, we conducted LASSO regression analysis of 23 genes. When log lambda ranged from -1.0 to -3.0, three genes were selected. A set of three mRNA (GAST, GPX3, THBS2) was found to promote the risk of live birth failure (Fig. 3) while CXCR4, DHRS3, DPP4, HABP2, HEY2, IGFBP1, LEPREL, MAP2K6, PROM1, SERPING, SFRP4, TIMP3, TNFAIP2, MUC1, HOXA10, GPR110, LIF, L-selectin, FKBP52, HAND2 were not selected. GAST was negatively associated with live birth. Along with the increased GPX3 expression levels in endometrium during the window of implantation, less women had live birth. The expression of THBS2 also had negative impact on live birth during window of implantation. ROC curves indicated that each of three genes had favorable predictive and prognostic accuracy. AUCs for GAST, GPX3, THBS2 reached to 0.736, 0.672, and 0.678, respectively (Fig. 4). The predictive model was established by adding the gene expression level and relative coefficient of each gene of the three genes. According to the coefficient value from LASSO analysis of each gene (Table 2), the risk scoring formula was calculated as follows: Risk score = (-0.063* GAST) + (-0.06*GPX3) + (-0.244*THBS2). Compared with the single gene, this gene model can achieve the highest accuracy of 0,772.

**Table 2:** Three genes and corresponding coefficient value in predicting live birth

mRNA	Coefficient
GAST	-0.063
GPX3	-0.06
THBS2	-0.244
Risk signature	Live birth

### Construction of risk score model based on gene and clinical features

**Fig. 5:**
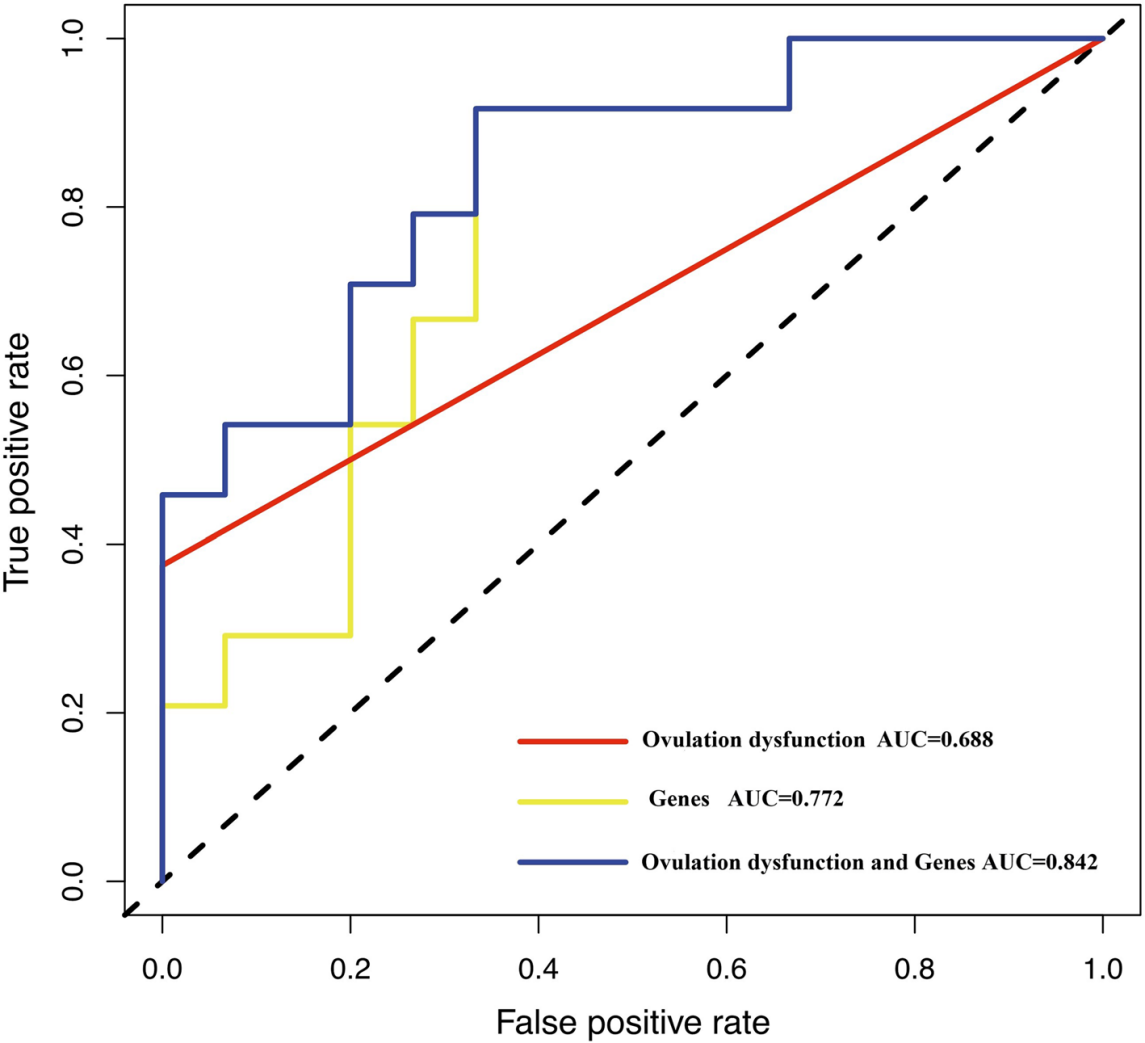
Comparison of predictive models for live birth. Risk model of clinical and gene features can achieve the highest accuracy (AUC = 0.842) compared with combined genes model (AUC = 0.772) and ovulation dysfunction model (AUC = 0.688) in predicting live birth

Since both clinical and gene features had its significance in live birth, ovulation dysfunction, GAST, GPX3, THBS2 were used to construct the final predictive model. According to ROC curves of the final model, AUC can reach to 0.842. However, AUC of ovulation dysfunction is 0.688, AUC of gene risk model is 0.772. As is shown in Fig. 5, the model of clinical and gene features can achieve the highest accuracy. According to the coefficient value from multivariate logistical regression analysis of each factor (Table 3), the risk scoring formula was calculated as follows: Risk score = (9.6 × 10^–10^*ovulation dysfunction) + (0.955* GAST) + (0.938*GPX3) + (0.839*THBS2).

**Table 3:** Clinical and three gene factors and corresponding coefficient value in predicting live birth

mRNA	Coefficient
ovulation dysfunction	9.6 × 10^–10^
GAST	0.955
GPX3	0.938
THBS2	0.839
Risk signature	Live birth

## Discussion

This prospective nested case–control study is, to our knowledge, the first study reporting on combined clinical factors and mRNA differential expression during the window of implantation between patients with live birth and not live birth, leading to the development of a prediction model to predict the chances of live birth. The model has modest discriminating power but excellent calibration. We used data that were prospectively collected in consecutive IVF patients who received morphologically good-quality frozen embryo transfer. The best model contains four independently significant predictors: ovulation dysfunction, GAST, GPX3, THBS2.

The human endometrial transcriptome has been extensively studied in a search of identifying diagnostic markers of the receptive endometrium and to provide more understanding into the complex regulation of endometrial functions. In our previous study, we uncovered 16 most significant up-regulated and downregulated genes that are involved in endometrial receptivity by mRNA microarray. Those genes were used, along with seven genes which were proved to be crucial in endometrial receptivity or may be effective in pathways related to receptive endometrium in other studies, were analyzed by lasso regression analysis to figure out the most valid transcriptomic signature. In 2011, ERA (endometrial receptivity analysis), a molecular diagnostic tool for the assessment of endometrium receptive or nonreceptive status has been used to analysis 238 target genes [11]. It has been used in clinical utility for a decade. In this study, genes in the final model overlapped with genes in the ERA. In 2020, transcriptomic signature with respect to human endometrial receptivity in Chinese women were developed, and our genes were all contained. Previous transcriptomic studies of human endometrium primarily focused on pre-receptive and receptive stages during the development of the luteal phase, and they revealed hundreds of simultaneously upregulated and downregulated genes that are involved in endometrial receptivity. However, the overlaps among different studies were relatively small, and a meta-analysis identified a meta-signature of endometrial receptivity involving 57 genes as putative receptivity markers [12]. Glycodelin and glutathione peroxidase 3 (GPX3) was included in this gene list and was confirmed in cell populations analyzed. Glycodelin is a glycoprotein from the human lipocalins superfamily and is mainly expressed in reproductive tissues: amniotic fluid [13], endometrium, and decidua [14]. In human endometrium, GPX3 was both upregulated in the stromal and epithelium. It has been identified as putative biomarker of endometrial receptivity in previous data mining and review studies. Expression of the GPX3 genes increases during mid-secretory phase coincident with the opening of this window [15]. Decreased midluteal glycodelin and GPX3 expressions have been reported in connection with some luteal phase deficiency [16]. It is also the most significant differentially expressed gene in ERA, with fold change 35.49 [11]. In this study, the expression of GPX3 was lower in patients with live birth compared with those who did not have live birth. We assume that the invasion of embryo needs cell damage, similar to the invasion of cancer cells. Gastrin (GAST) is a peptide hormone that stimulates secretion of gastric acid, is secreted from the G cells in the stomach. Its main function is to stimulate secretion of hydrochloric acid by the gastric mucosa, which results in gastrin formation inhibition. It is involved in positive regulation of cell population proliferation and is responsible for hormone activity. During the receptive window, proliferation of epithelial was inhibited, stroma cells decidualized. In this study, GAST was negatively associated with live birth, consistent with the fact that the receptive endometrium has decreased proliferation and increased decidualization. Thrombospondin 2 (THBS2) belongs to the thrombospondin family, which is a disulfide-linked homotrimeric glycoprotein that mediates cell-to-cell and cell-to-matrix interactions. The protein encoded by THBS2 has been shown to function as a potent inhibitor of tumor growth and angiogenesis. Studies of the mouse counterpart suggest that this protein may modulate the cell surface properties of mesenchymal cells and be involved in cell adhesion and migration. THBS2 is broadly expressed in endometrium, and is a disulfide-linked homotrimer glycoprotein that mediates cell-to-cell and cell-to-matrix interactions. This protein has been shown to function as a potent inhibitor of angiogenesis. blastocyst invasion to the endometrial epithelium and the subsequent invasion of the maternal tissue is an essential element of embryo implantation [12]. Angiogenesis is particularly important for implantation and placenta formation. Down regulation of THBS2 during WOI the in receptive endometrium emphasized importance of angiogenesis.

Variables significant in a 15-year period model for live birth prediction were used as clinical variables in this study. However, none was qualified as a predictor of live birth after IVF in patients with MGE except for ovulation dysfunction. Ovulation dysfunction is one of the indications for IVF. In this study, nine patients (23.1%) had ovulation dysfunction, including three luteinized unmatured follicle syndromes (LUFS), two polycystic ovarian syndromes (POCS), three diminished ovarian reserve (DOR), one premature ovulation and ovarian endometrioma. Patients with ovulation dysfunction was positively correlated with live birth after morphology good embryo transfer. As in earlier studies, infertility indication qualified as an independent predictor, which was mainly because of the low success rates associated with tubal subfertility. As for ovulation dysfunction, which includes lots of diseases, its association with live birth remains controversial. Anovulation, including women with polycystic ovary syndrome, proved to be negatively related to live birth [17]. While endometriosis was not associated with a reduced prognosis, including those with a visible ovarian endometrioma [18–20]. In our study, all patients received MGE transfer, suggesting that patients with ovulation dysfunction tend to have a higher rate of good endometrial receptivity. When this cause was properly treated, patients may have higher rate of good reproductive outcomes. Tubal subfertility may have inflammatory cytokines which could influence endometrium receptivity.

All genes were derived during the window of implantation, revealing the gene characteristics of the receptive endometrium compared with unreceptive endometrium. Genes that were more associated with invasion and hyperplasia were favorable to live birth. Genes upregulated in the malignant tumor were also upregulated in the receptive endometrium. The implantation of the embryo may share similar biological features as cancer. The ability to activate procession on endometrium allows embryo invasion, cell damage, and vascular remodeling. The information could be used in the development of a model to decide which patients have better receptivity and which patients have higher live birth rate when retrieving high-quality embryos.

The discriminative capacity of the model was modest due to the limited patients enrolled. The test for calibration is needed in the future study. However, it offers an attempt to reveal gene during WOI associated with live birth. This may also bring up a direction of other medical centers to conduct lager trial to assess model accuracy using cross-validation approaches. There is also another limitation of our study. The real process of implantation is a communication between the embryo and the endometrium, which is inaccessible and cannot be observed directly. Only in the presence of an embryo, some genes of endometrium were up and down regulated, and the biopsy without an embryo in our study could not reflect the real changes of gene expression in the endometrium completely. However, the scarcity of human embryos and ethical problems make this process difficult to investigate. In vitro models combining human embryos and primary endometrial epithelial cells [21] or Ishikawa cells [22] have provided evidence for the embryonic regulation of endometrial epithelial cell surface molecules and signaling pathways required for successful apposition and attachment in the process of implantation. Using TE-spheroids, Vergaro et al. showed that TE attachment induced a wave of transcriptional changes in the endometrial epithelium [23]. Recent research of in vitro blastoids-endometrial organoids model could replicate the directional process of implantation with a higher efficiency [24], which provides a new useful tool for the ongoing study.

## Conclusion

In conclusion, in this prospective nested case–control study, we identified three transcriptions during the receptive endometrium to predict the live birth of patients with MGE. Furthermore, we constructed a prediction model for live birth with both transcription signature and clinical features. ovulation dysfunction, GAST, GPX3, THBS2 were predictors of live birth. Compared with the transcription signature model, this model combined with both clinical transcription signature has the highest AUC.

### Supplementary Information

**Additional file 1: Supplemental table 1.** Sequences of mRNAs primers used for real-time PCR amplification. **Supplemental table 2.** Fold change of mRNA under real-time PCR in all patients. **Supplemental table 3.** Univariate Logistic Regression Analysis between live birth and clinical features.

## Data Availability

The datasets generated and/or analysed during the current study are not publicly available due to policy of Peking University People's Hospital, but are available from the corresponding author on reasonable request.
