# Supplementary material for: A combined clinical and specific genes’ model to predict live birth for in vitro fertilization and embryo transfer patients
Source: BMC Pregnancy Childbirth. 2023 Sep 30;23:702. doi: 10.1186/s12884-023-05988-6 (PMC10541716; doi:10.1186/s12884-023-05988-6)
Supplement: Supplementary file 1 — Additional file 1: Supplemental table 1. Sequences of mRNAs primers used for real-time PCR amplification. Supplemental table 2. Fold change of mRNA under real-time PCR in all patients. Supplemental table 3. Univariate Logistic Regression Analysis between live birth and clinical features. [file 12884_2023_5988_MOESM1_ESM.docx]

Supplemental table 1. Sequences of mRNAs primers used for real-time PCR amplification

| mRNAs | Primers |
| --- | --- |
| CXCR4 | F: AGATAACTACACCGAGGAAATGG  R: GGGAAGCGTGATGACAAAGAG |
| DHRS3 | F: CTGAAGGAGACGACGGAGGAGAT  R: TGTTGGGACTTGAGGAGGGCAT |
| DPP4 | F: CCATACAAATCACTGCTCCTGCTTCT  R: TGACCGAATAGTTCTGAATCCTCCTGA |
| GAST | F: TGATCTTTGCACTGGCTCTGGC  R: TGCTTCTTGGACGGGTCTGC |
| GPX3 | F: TGATCTTTGCACTGGCTCTGGC  R: TGCTTCTTGGACGGGTCTGC |
| HABP2 | F: ACGAGCAAGCAGTCAGCACAG |
|  | R: CAGGCAGATGACACGCAGGTT |
| HEY2 | F: CAAGAAAGAAAAGGAGAGGGATT  R: GCGTGTGCGTCAAAGTAGC |
| IGFBP1 | F: AGCACGGAGATAACTGAGGAGGAG  R: CCTTGGCTAAACTCTCTACGACTCTG |
| LEPREL | F: TTGGATCAGATATGGAGGACG  R: CGAGTTGTAGACGAATGTGATGTT |
| MAP2K6 | F: GGATTGTTGCTGGACTTTGGA  R: GGTTTGGCTTTTTGATGGGTT |
|  |  |
| PROM1 | F: CACTTACGGCACTCTTCACCT  R: TGTCTATTCCACAAGCAGCAAA |
|  |  |
| SERPING | F: AGTGACGCCAACTTGGAGCT  R: CAGGTAGATAGCATTGAGGAGGAC |
|  |  |
| SFRP4 | F: CCTCATCACCCATCCCTCG |
|  | R: TCTTCTTGTCCTGAACTGTTCTCC |
| THBS2 | F: CCTACATCTCCAACGCCAACCAG |
|  | R: CCATCACCGTCCAAGTCCTCCT |
| TIMP3 | F: CAACAAGTACCAGTACCTGCTGAC |
|  | R: GAGACACTCGTTCTTGGAAGTCAC |
| TNFAIP2 | F: AACAACTGCCTGTCCTTCCG |
|  | R: TCGTGTCTACAGTGGCGATG |
| MUC1 | F: CTGAATCTGTTCTGCCCCCT  R: TTAGGGGCTGTGGTAGCTGTA |
|  |  |
| HOXA10 | F: CGGGAGTTCTTCTTCAGCGT |
|  | R: GGGCTATCTCCATGCATCCC |
| GPR110 | F: GTCACGCAACCTAGCAATACCTAC  R: AAGTTCACAGCCACAATAGCCAGT |
| LIF | F: GCCACCCATGTCACAACAAC |
|  | R: GCCACATAGCTTGTCCAGGT |
| L-selectin | F: GAGCTGGGTACCATGGACTG |
|  | R: CCACTGGAATGAAGAGGGGG |
| FKBP52 | F: GGAGATGAAGGCGACCGAGA |
|  | R: TGCGAATGATTCCGCCATCT |
| HAND2 | F: AGAGGAAGAAGGAGCTGAACG |
|  | R: CTTTTCCGGAGTCCTGGGTC |

Supplemental table 2. Fold change of mRNA under real-time PCR in all patients

| Sample | **CXCR4** | **DHRS3** | **DPP4** | **GAST** | **GPX3** | **HABP2** | **HEY2** | **IGFBP1** | **LEPREL** | **MAP2K6** | **PROM1** | **SERPING** | **SFRP4** | **THBS3** | **TIMP3** | **TNFAIP2** | **MUC1** | **HAND2** | **HOXA10** | **GPR110** | **LIF** | **L-selectin** | **FKBP52** |
| --- | --- | --- | --- | --- | --- | --- | --- | --- | --- | --- | --- | --- | --- | --- | --- | --- | --- | --- | --- | --- | --- | --- | --- |
| 1 | 0.18 | 0.21 | 0.15 | 0.06 | 0.07 | 0.04 | 1.6 | 0.07 | 0.75 | 2.81 | 0.4 | 0.35 | 1.34 | 0.87 | 1.07 | 0.33 | 7.38 | 0.32 | 1.07 | 0.48 | 0.02 | 0.35 | 0.31 |
| 2 | 3.16 | 3.53 | 12.08 | 8.06 | 19.71 | 3.25 | 0.39 | 6.03 | 0.62 | 0.12 | 2.48 | 1.85 | 0.32 | 0.61 | 0.64 | 2.36 | 0.32 | 0.79 | 0.45 | 0.73 | 3.20 | 27.37 | 0.42 |
| 3 | 1.19 | 2.64 | 17.56 | 7.46 | 13.94 | 5.29 | 0.58 | 25.66 | 2.22 | 0.76 | 1.92 | 1.81 | 0.92 | 2.02 | 3.59 | 1.08 | 0.11 | 0.50 | 1.04 | 0.49 | 1.22 | 0.90 | 0.20 |
| 4 | 1.48 | 2.97 | 1.28 | 11.63 | 7.31 | 8.35 | 0.85 | 4.32 | 2.68 | 1.35 | 1.31 | 2.92 | 0.98 | 7.14 | 1.56 | 2.34 | 0.41 | 1.26 | 0.91 | 0.53 | 1.26 | 1.39 | 2.93 |
| 5 | 1.44 | 3.07 | 7.03 | 12.21 | 8.29 | 2.07 | 0.36 | 1.19 | 2.13 | 0.14 | 1.22 | 2.54 | 0.48 | 2.24 | 3.47 | 1.92 | 0.21 | 1.63 | 1.28 | 0.41 | 1.63 | 0.49 | 0.31 |
| 6 | 0.61 | 0.88 | 0.34 | 2 | 0.53 | 3.56 | 0.68 | 2.68 | 1.92 | 1.48 | 1.03 | 0.98 | 0.47 | 4.07 | 1.69 | 0.58 | 9.38 | 0.21 | 0.18 | 0.88 | 0.58 | 0.34 | 1.83 |
| 7 | 0.96 | 4.72 | 42.35 | 15.45 | 32.02 | 3.76 | 0.34 | 120.37 | 4.32 | 0.5 | 1.44 | 2.73 | 0.85 | 3.15 | 1.59 | 1.26 | 0.36 | 1.22 | 1.00 | 0.50 | 2.33 | 3.34 | 11.15 |
| 8 | 1.68 | 2.08 | 0.53 | 1.42 | 1.36 | 0.93 | 1.17 | 1.14 | 1.82 | 1.01 | 0.51 | 1.2 | 2.1 | 1.56 | 1.94 | 1.03 | 4.14 | 0.30 | 1.13 | 0.46 | 0.39 | 0.91 | 0.28 |
| 9 | 3.03 | 2.13 | 2.02 | 4.76 | 4.23 | 4.87 | 1.71 | 0.31 | 1.04 | 1.11 | 0.62 | 3.18 | 2.05 | 1.37 | 1.51 | 3.43 | 11.23 | 0.95 | 1.72 | 0.30 | 1.77 | 1.32 | 0.58 |
| 10 | 2.77 | 4.92 | 12.59 | 9.85 | 12.56 | 11.57 | 0.25 | 21.58 | 2.87 | 1.24 | 1.73 | 2.94 | 0.32 | 5.19 | 2.27 | 3.05 | 2.37 | 1.04 | 0.94 | 0.52 | 1.55 | 3.25 | 33.01 |
| 11 | 4.02  0.96 | 0.6 | 0.15 | 12.38 | 0.54 | 17.91 | 0.06 | 1.39 | 1.31 | 5.86 | 0.07 | 1.15 | 0.03 | 6.99 | 0.38 | 0.28 | 0.72 | 0.12 | 0.59 | 0.67 | 0.78 | 4.69 | 43.68 |
| 12 |  | 0.99 | 0.78 | 0.01 | 0.14 | 0.11 | 2 | 10.9 | 1.87 | 1.89 | 1.97 | 0.26 | 11.94 | 0.72 | 0.65 | 0.42 | 0.17 | 0.42 | 3.16 | 0.11 | 0.04 | 0.51 | 0.45 |
| 13 | 0.88  0.22 | 1.12 | 1.16 | 6.59 | 2.68 | 0.58 | 1.35 | 1.28 | 0.87 | 1.13 | 0.5 | 1.07 | 0.52 | 0.86 | 1.13 | 0.93 | 8.38 | 0.30 | 1.20 | 0.43 | 0.42 | 0.65 | 0.69 |
| 14 |  | 4.82 | 0 | 5.28 | 16.92 | 7.79 | 0.28 | 39.43 | 3.89 | 0.47 | 1.46 | 2.8 | 0.21 | 9.55 | 3.4 | 1.26 | 2.52 | 0.36 | 0.05 | 0.97 | 1.42 | 0.22 | 1.44 |
| 15 | 0.38  2.1 | 0.48 | 0.36 | 0.72 | 0.32 | 0.4 | 1.77 | 0.14 | 1.1 | 2.95 | 1.01 | 0.46 | 1.36 | 0.57 | 0.97 | 0.55 | 0.21 | 0.65 | 1.86 | 0.28 | 0.06 | 0.51 | 0.51 |
| 16 |  | 3.07 | 3.02 | 11.88 | 9.92 | 2.37 | 0.85 | 13.28 | 0.62 | 0.34 | 2.48 | 2.14 | 0.68 | 1.5 | 0.97 | 1.83 | 13.49 | 0.79 | 0.87 | 0.55 | 1.85 | 1.19 | 0.82 |
| 17 | 0.41 | 0.42 | 0.27 | 4.32 | 0.64 | 1.16 | 0.74 | 0.76 | 0.75 | 0.52 | 0.91 | 0.87 | 0.74 | 3.06 | 1.32 | 1.23 | 0.22 | 0.88 | 1.37 | 0.39 | 0.08 | 0.25 | 0.34 |
| 18 | 0.59 | 0.23 | 0.12 | 0.01 | 0.02 | 0.09 | 2.05 | 0.06 | 0.62 | 1.28 | 0.47 | 0.25 | 0.94 | 0.29 | 0.47 | 0.35 | 0.23 | 0.47 | 1.18 | 0.44 | 0.02 | 0.47 | 0.41 |
| 19 | 0.11 | 0.27 | 0.17 | 0.19 | 0.12 | 0.19 | 1.14 | 0.13 | 0.41 | 2.3 | 0.34 | 0.34 | 0.75 | 0.3 | 0.52 | 0.21 | 0.10 | 0.14 | 0.53 | 0.69 | 0.23 | 1.08 | 5.93 |
| 20 | 3.07 | 2.1 | 2.35 | 15.56 | 7.73 | 3.82 | 0.66 | 1.96 | 1.39 | 0.22 | 1.32 | 2.07 | 0.46 | 1.75 | 1.26 | 1.55 | 0.25 | 1.23 | 1.48 | 0.36 | 1.11 | 0.39 | 0.32 |
| 21 | 1.79 | 1.17 | 2.02 | 0.09 | 0.58 | 0.67 | 0.63 | 73.08 | 0.43 | 2.13 | 11.09 | 0.53 | 0.12 | 0.26 | 0.64 | 2.38 | 0.24 | 0.23 | 0.15 | 0.90 | 0.17 | 1.02 | 1.80 |
| 22 | 0.9 | 0.77 | 0.83 | 1.39 | 2.13 | 0.97 | 1.52 | 0.18 | 1.25 | 1.5 | 0.56 | 1.29 | 1.62 | 1.13 | 1.78 | 1.59 | 0.28 | 0.70 | 2.71 | 0.15 | 0.34 | 0.66 | 0.55 |
| 23 | 0.46 | 0.33 | 0.26 | 2.3 | 0.49 | 1.63 | 1.16 | 0.52 | 0.7 | 1.4 | 0.81 | 0.85 | 0.65 | 1.01 | 0.7 | 0.39 | 0.17 | 0.24 | 0.21 | 0.87 | 0.13 | 0.20 | 1.97 |
| 24 | 0.37 | 0.14 | 0.18 | 0.21 | 0.01 | 0.74 | 1.32 | 10.9 | 0.47 | 1.99 | 0.23 | 0.22 | 2.33 | 0.24 | 0.46 | 0.18 | 0.12 | 0.13 | 0.28 | 0.83 | 0.09 | 0.14 | 1.05 |
| 25 | 0.42  2.95 | 0.2 | 0.21 | 0.09 | 0.06 | 0.03 | 1.2 | 0.07 | 1.05 | 3.39 | 0.4 | 0.55 | 1.04 | 0.33 | 1.05 | 0.52 | 0.24 | 0.64 | 1.87 | 0.27 | 0.03 | 0.65 | 0.57 |
| 26 |  | 4.47 | 4.1 | 1.49 | 14.63 | 10.14 | 1.05 | 3.23 | 1.53 | 0.76 | 4.06 | 3.27 | 1.12 | 2.72 | 2.27 | 3.86 | 0.34 | 1.18 | 1.46 | 0.36 | 0.97 | 0.61 | 0.40 |
| 27 | 1.07 | 0.62 | 0.18 | 4 | 0.35 | 0.63 | 1.06 | 0.59 | 1.19 | 1.06 | 0.54 | 0.96 | 0.4 | 1.47 | 1.35 | 0.99 | 7.31 | 0.92 | 1.19 | 0.44 | 0.10 | 0.41 | 0.33 |
| 28 | 1.96 | 2.97 | 6.56 | 15.45 | 10.42 | 3.32 | 0.46 | 33.16 | 3.32 | 0.15 | 1.17 | 4.28 | 0.61 | 4.3 | 2.54 | 1.66 | 0.16 | 1.46 | 1.67 | 0.32 | 1.52 | 1.75 | 0.31 |
| 29 | 3.34 | 2.31 | 3.42 | 7.36 | 7.57 | 2.35 | 0.47 | 5.86 | 1.87 | 0.21 | 1.37 | 1.76 | 0.67 | 2.47 | 1.43 | 2.16 | 0.52 | 1.40 | 1.79 | 0.29 | 1.66 | 0.70 | 0.49 |
| 30 | 3.63 | 6.18 | 26.07 | 22.78 | 34.08 | 5.67 | 0.5 | 29.89 | 3.25 | 0.16 | 3.18 | 2.75 | 0.8 | 2.45 | 1.46 | 2.67 | 0.22 | 1.15 | 1.60 | 0.33 | 3.82 | 1.41 | 0.49 |
| 31 | 0.73 | 0.63 | 0.44 | 0.1 | 0.16 | 0.25 | 1.66 | 8.35 | 1.4 | 1.47 | 0.9 | 0.33 | 1.71 | 0.75 | 0.84 | 0.66 | 13.66 | 0.52 | 1.80 | 0.29 | 0.09 | 0.57 | 1.08 |
| 32 | 1.2 | 4.37 | 9.22 | 11.63 | 8.88 | 4.21 | 0.63 | 1.5 | 1.89 | 0.81 | 1.85 | 1.64 | 1.3 | 1.46 | 0.78 | 1.33 | 16.21 | 0.30 | 0.13 | 0.91 | 1.82 | 0.21 | 0.55 |
| 33 | 4.56 | 8.39 | 19.22 | 9.71 | 28.26 | 10.14 | 0.91 | 1.49 | 1.52 | 0.26 | 5.39 | 4.56 | 2.92 | 2.29 | 1.34 | 1.92 | 0.13 | 0.50 | 1.66 | 0.32 | 3.43 | 1.34 | 0.38 |
| 34 | 1.2 | 1.29 | 2.02 | 9.19 | 3.58 | 2.78 | 0.7 | 1.74 | 0.56 | 0.76 | 0.83 | 1.25 | 0.85 | 0.79 | 0.7 | 0.7 | 0.27 | 0.15 | 0.16 | 0.89 | 0.57 | 0.16 | 0.99 |
| 35 | 2.34 | 5.97 | 7.91 | 4.5 | 13 | 11.57 | 2.1 | 0.46 | 0.93 | 0.69 | 5.78 | 2.48 | 2.19 | 1.16 | 0.33 | 2.11 | 0.46 | 0.31 | 1.22 | 0.43 | 1.96 | 0.58 | 0.52 |
| 36 | 2.3 | 4.05 | 4.77 | 3.43 | 9.39 | 5.29 | 0.53 | 2.09 | 1.4 | 0.2 | 1.66 | 2.26 | 0.39 | 1.64 | 1.11 | 2.66 | 0.23 | 1.09 | 1.63 | 0.32 | 1.83 | 0.82 | 0.54 |
| 37 | 0.18 | 0.18 | 0.17 | 0.1 | 0.06 | 0.02 | 2.25 | 0.16 | 0.56 | 3.01 | 0.29 | 0.4 | 1.25 | 0.36 | 0.57 | 0.33 | 2.07 | 0.05 | 0.78 | 0.58 | 0.02 | 0.70 | 0.17 |
| 38 | 0.26 | 0.16 | 0.17 | 0.05 | 0.04 | 0.07 | 1.25 | 0.04 | 0.37 | 1.95 | 0.27 | 0.27 | 0.96 | 0.14 | 0.58 | 0.33 | 30.32 | 0.21 | 1.25 | 0.42 | 0.02 | 0.30 | 0.32 |
| 39 | 1.5 | 0.83 | 1.14 | 1.38 | 2.68 | 1.87 | 1.63 | 1.54 | 1.06 | 2.03 | 1 | 1.21 | 2.53 | 1.11 | 1.4 | 1.24 | 0.25 | 0.37 | 1.70 | 0.31 | 0.29 | 1.53 | 0.54 |

Supplemental table 3. Univariate Logistic Regression Analysis between live birth and clinical features

|  | Value | OR (95%CI) | P-value |
| --- | --- | --- | --- |
| **Age (year)** | 32.05 ± 4.30 | 1.03 (0.88, 1.20) | 0.71 |
| **Body mass index (kg/m^2^)** | 23.21 ± 2.83 | 0.99 (0.79, 1.24) | 0.92 |
| **Fertility history** |  |  |  |
| Duration of infertility, y (IQR) | 3.14 ± 2.38 | 1.04 (0.78, 1.39) | 0.77 |
| Primary infertility, no. (%) | 24 (61.54%) | 1.75 (0.47, 6.57) | 0.41 |
| **Indications for IVF, no. (%)** |  |  |  |
| Tubal factor | 19 (48.72%) | 1.14 (0.31, 4.16) | 0.84 |
| Male factor | 29 (74.36%) | 1.09 (0.25, 4.75) | 0.91 |
| Ovulatory factor | 9 (23.08%) | 4.000(1.13−8.91) | 0.018^*^ |
| **Number of failed** **embryo transfer cycle** |  |  |  |
| 0 | 25 (64.10%) | 1.0 |  |
| 1 | 5 (12.82%) | 0.84 (0.12, 6.03) | 0.87 |
| 2 | 4 (10.26%) | 1.69 (0.15, 18.71) | 0.67 |
| 3 | 3 (7.69%) | 1.12 (0.09, 14.20) | 0.93 |
| 4 | 1 (2.56%) | 0.00 (0.00, Inf) | 0.99 |
| 8 | 1 (2.56%) | 0.00 (0.00, Inf) | 0.99 |
| **Repeated implantation failure** | 5 (12.82%) | 0.36 (0.05, 2.49) | 0.30 |
| **Hormone test and Ultrasound** |  |  |  |
| AMH, ng/ml | 4.19 ± 2.58 | 0.91 (0.70, 1.17) | 0.44 |
| Antral Follicle Counting | 13.85 ± 6.17 | 1.00 (0.90, 1.11) | 0.99 |
| Ovarian sensitivity index | 0.70±0.31 | 0.55 (0.06,4.84) | 0.59 |
| **COH protocol** |  |  |  |
| GnRH agonists, no. (%) | 23 (58.97%) | 1.88 (0.48, 7.26) | 0.36 |
| GnRH antagonists, no. (%) | 14 (35.90%) | 1.0 |  |
| CC Mild stimulation, no. (%) | 2 (5.13%) | inf. (0.00, Inf) | 0.99 |
| **Duration of COH (day)** | 10.59 ± 1.80 | 0.96 (0.67, 1.38) | 0.83 |
| **Total dose of FSH/hMG (UI)** | 2506.09 ± 847.49 | 1.00 (1.00, 1.00) | 0.3 |
| **No. of oocytes retrieved** | 13.38 ± 5.99 | 0.97 (0.87, 1.08) | 0.61 |
| **Endometrium thickness before embryo transfer, mm** | 9.55 ± 2.12 | 1.32 (0.93, 1.87) | 0.12 |
| **Regiment of endometrial preparation** |  |  |  |
| Natural cycle no. (%) | 24(61.5%) | 1.11(0.3, 4.17) | 0.88 |
| Artificial cycle no. (%) | 15(38.5%) | 1.0 |  |
| **Type of embryo transferred** |  |  |  |
| Cleavage transfer, no. (%) | 17(43.6) | 1.0 |  |
| Blastocyst transfer, no. (%) | 22(56.4) | 0.31(0.08, 1.25) | 0.10 |

Note: IVF: In vitro fertilization; AMH: anti-Mullerian hormone; COH: controlled ovarian hyperstimulation; FSH: follicle stimulating hormone; hMG: human menopausal gonadotropin
